# Quantifying Condition-Dependent Intracellular Protein Levels Enables High-Precision Fitness Estimates

**DOI:** 10.1371/journal.pone.0075320

**Published:** 2013-09-25

**Authors:** Kerry A. Geiler-Samerotte, Tatsunori Hashimoto, Michael F. Dion, Bogdan A. Budnik, Edoardo M. Airoldi, D. Allan Drummond

**Affiliations:** 1 Department of Biochemistry and Molecular Biology, University of Chicago, Chicago, Illinois, United States of America; 2 Center for Genomics and Systems Biology, Department of Biology, New York University, New York, New York, United States of America; 3 Department of Statistics, Harvard University, Cambridge, Massachusetts, United States of America; 4 FAS Center for Systems Biology, Harvard University, Cambridge, Massachusetts, United States of America; 5 The Broad Institute of Harvard & MIT, Cambridge, Massachusetts, United States of America; Texas A&M University, United States of America

## Abstract

Countless studies monitor the growth rate of microbial populations as a measure of fitness. However, an enormous gap separates growth-rate differences measurable in the laboratory from those that natural selection can distinguish efficiently. Taking advantage of the recent discovery that transcript and protein levels in budding yeast closely track growth rate, we explore the possibility that growth rate can be more sensitively inferred by monitoring the proteomic response to growth, rather than growth itself. We find a set of proteins whose levels, in aggregate, enable prediction of growth rate to a higher precision than direct measurements. However, we find little overlap between these proteins and those that closely track growth rate in other studies. These results suggest that, in yeast, the pathways that set the pace of cell division can differ depending on the growth-altering stimulus. Still, with proper validation, protein measurements can provide high-precision growth estimates that allow extension of phenotypic growth-based assays closer to the limits of evolutionary selection.

## Introduction

The close link between growth rate and fitness, coupled with the sensitivity of growth to genetic and environmental perturbations, has made growth rate among the most-studied phenotypes in biology. Studies of growth have provided evidence for the functions of specific genes, when knockouts halt or slow growth in some environmental conditions but not others [1,2], and have uncovered programs of responses to diverse conditions such as nutrient limitation, exposure to antibiotics, heat, or osmotic shock [3-5]. However, most techniques can reliably distinguish growth rate differences only on the order of 10%–20% [3]. Moreover, laboratory perturbations generating fitness effects large enough to measure by these techniques may induce a general stress response [6,7] or another response that confounds perturbation-specific effects on growth. More precise growth rate measurements require substantial cell culturing, frequent dilution, high replicate numbers, and many generations of exponential growth. The most precise techniques can distinguish growth rates differences as small as 0.5–1% [2,4,8].

This precision pales in comparison to that of natural selection, which can efficiently distinguish between lineages whose growth rate differs by roughly the inverse of the effective population size, which for many animals exceeds 10^5^ and for microbes can exceed 10^8^. For example, a mutation causing a fitness defect of 0.01% in popular microbial model organisms such as *E. coli* or budding yeast will be undetectable in the laboratory using any present-day method, but is virtually certain to be evolutionarily lethal to the mutant lineage. Weak selection on traits conferring tiny fitness differences contributes critically to evolutionary variation, underlying widespread phenomena such as codon bias [9] and evolutionary rate differences between genes and genomic regions [10].

To extend the reach of empirical studies into milder fitness regimes will require substantial improvements and, likely, nontraditional approaches. One strategy is exemplified by the recent demonstration of hyper-precise measurements of the mass of single cells [11,12]; such approaches, however, are not well-suited to measuring differences in cell doubling time over large numbers of cells under arbitrary growth conditions, the typical aim of a fitness assay. In this study, we explore a fundamentally different method to quantifying cell growth that extracts instantaneous growth rate from instantaneous gene expression [13].

In budding yeast cultures with growth rate differences of 15% or greater, about a quarter of the genome is expressed in a growth-rate-dependent fashion [6,7]. Indeed, many regulatory responses appearing during cell stresses appear to be a secondary response to slowed growth rather than to any specific stress [6]. Growth-rate differences induced by heat shock or nutrient limitation in both batch and chemostat cultures have been reliably predicted by monitoring expression of these growth-dependent genes [6,13]. We reasoned that such a response to growth would provide an attractive, general signal that could be exploited for inferring smaller growth differences than can presently be measured by cell counting. Here, we probe the sensitivity and generality of this growth-rate prediction technique.

Building on previous efforts [14], we monitor protein abundances rather than transcript abundances. Using relative protein abundance measurements obtained via whole-proteome mass spectrometry, we search for proteins that demonstrate growth-dependent expression in strains with mild (1%) growth defects induced by protein misfolding. The levels of these proteins constitute a proteomic growth-rate “speedometer” that rivals other growth assays in precision, distinguishing growth rate differences of less than half a percent. Proteomic growth quantitation is especially promising given frequent advances in mass spectrometry that allow for greater sensitivity at lower cost [15] and higher throughput [16], while offering the opportunity to study growth-related phenomena that involve only post-transcriptional processes. However, we find that one growth model does not fit all studies. In fact, there is surprisingly little overlap between the growth-predictive proteins we detect and those previously found to be correlated with altered growth rate [6,7,14]. This result suggests that budding yeast’s response to growth rate depends on the growth-altering perturbation. It also offers the possibility that some subtle growth perturbations can be studied free of the confounding influence of a systemic growth-rate response.

## Results

**Figure 1:**
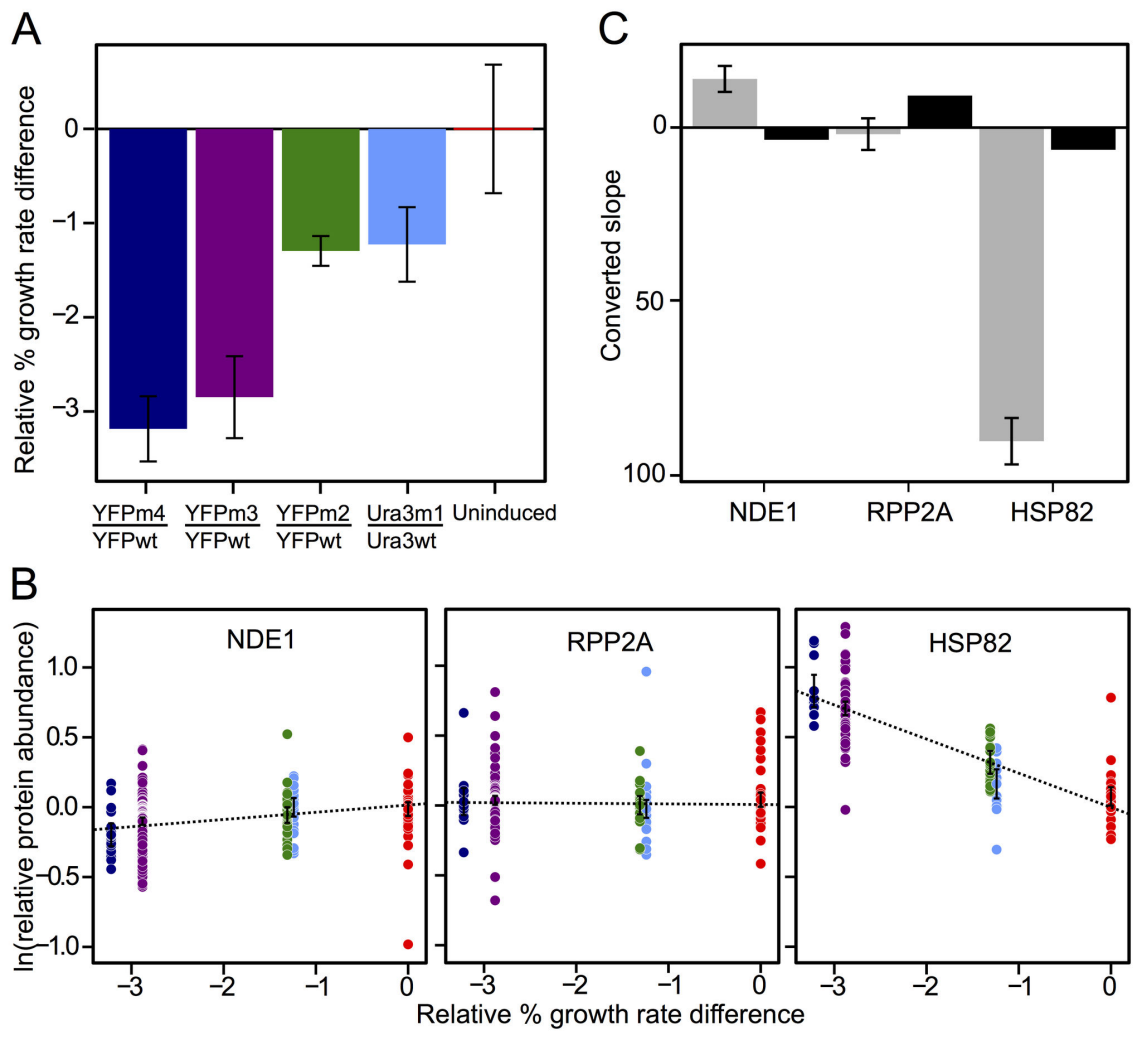
Constructing a proteomic growth model. (**A**) The average % growth rate difference relative to the ‘wt’ strain in each pair (figure produced using previously published data) [4]. Error bars show +/- one standard deviation. (**B**) Log relative protein abundance measurements vs. growth rate for 3 example proteins. ‘NDE1’ is repressed in strains with decreased growth rate. ‘RPP2A’ is unresponsive to changing relative growth rates. ‘HSP82’ is induced in strains with decreased growth rate. Colors correspond to strain pairs in (**A**). Error bars show +/- one standard deviation. (**C**) Slopes for three example proteins from (**B**) multiplied by a conversion factor (see Methods S1) to allow direct comparison between our slopes (across relative % growth differences; grey) and slopes found previously (across absolute growth differences; black) [6]. We use unconverted slopes to predict growth differences. Error bars display +/- standard error on the slope.

All of the growth perturbations we study were induced by intracellular protein misfolding, and have been quantified previously by competitive growth of paired strains in batch culture, monitoring relative cell counts by flow cytometry [4] to extract growth-rate differences (Figure **1A**). To monitor relative protein abundance between paired strains—each pair including one unperturbed strain (expressing a wild-type protein) and one growth-perturbed strain (expressing a misfolded variant of the same protein) — we used stable-isotope labeling of amino acids in cell culture (SILAC) [17], and quantified labeled/unlabeled protein ratios in 1:1 mixtures of total protein harvested from these paired strains during exponential growth (Table **S1**). A total of five pairs of strains are included in this study, including a control pairing in which both strains are uninduced (Table **1**).

**Table 1:** Proteomic datasets.

**Dataset ID**	**Proteins induced in paired strains**	**Fold difference in misfolded protein (m/wt**)	**Growth rate difference by competition assay (%**)	**Replicates**	**Proteins that pass filtering**
Uninduced	-	-	0	6	648
Ura3m1	Ura3wt or Ura3m1	n.d.	-1.24	4	502
YFPm2	YFPwt or YFPm2	6.95	-1.31	4	585
YFPm3	YFPwt or YFPm3	7.27	-2.88	7	2010
YFPm4	YFPwt or YFPm4	10.31	-3.22	2	497

All strains included in this experiment harbor a genomically integrated, galactose inducible, wild-type (“wt”) or misfolded (“m1–m4”) variant of either Ura3 (orotidine 5-phosphate decarboxylase) or YFP (yellow fluorescent protein). Misfolded protein variants were created in a previous study [4] in which the amount of misfolding relative to the wild-type protein was quantified. For the strain pairs we study, growth rate differences (induced by differences in intracellular protein misfolding) have been quantified previously by monitoring growth competitions using flow cyometry [4] and are relative to the wild-type protein expressing strain in each pair. “Uninduced” represents a control dataset in which neither a natively folded nor misfolded protein variant is expressed in either strain and no growth rate difference between strain pairs is expected. We collected proteomic data from a minimum of two replicate experiments for each dataset (Table **S1**). From each replicate, proteins for which relative abundance was measured at least four times are retained.

**Figure 2:**
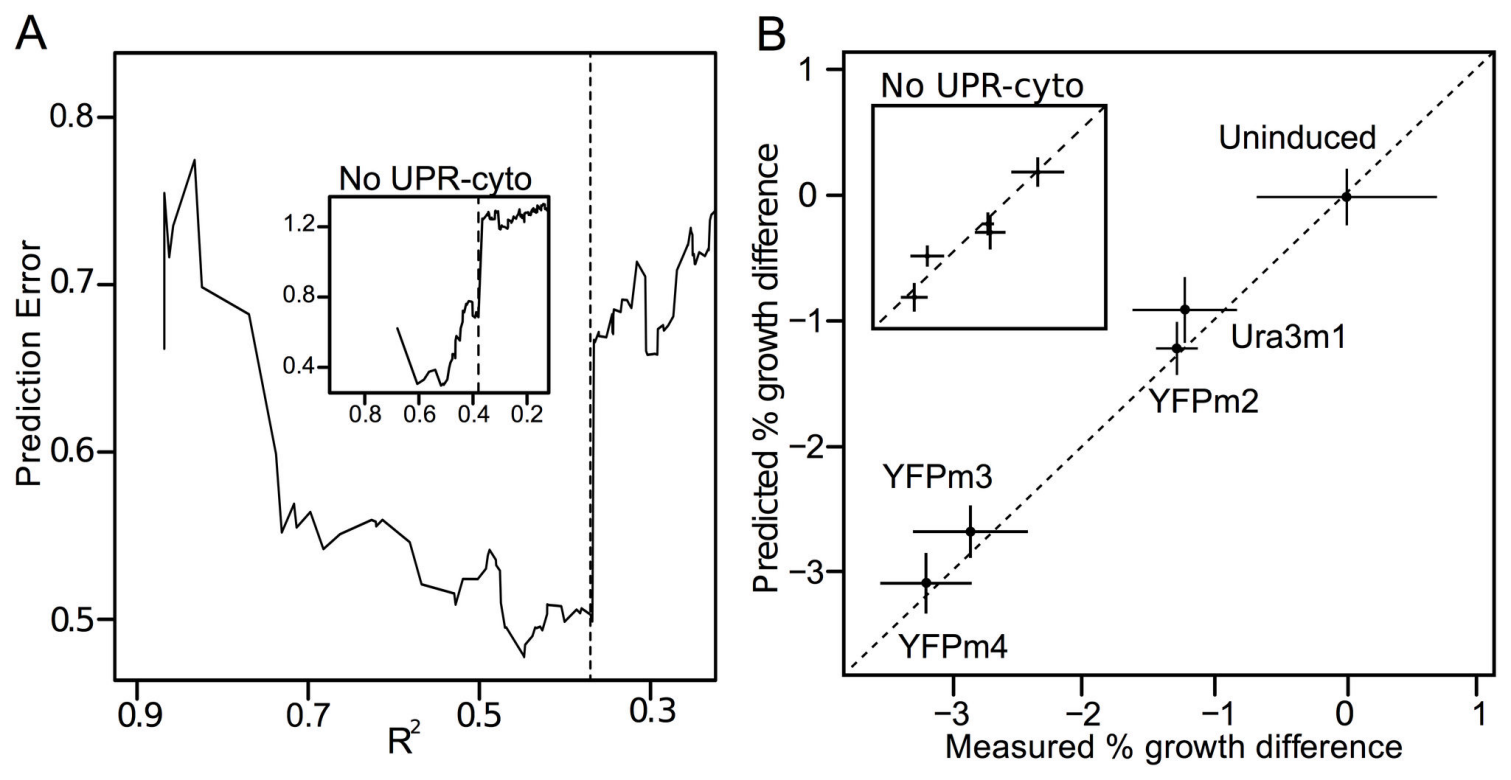
A proteomic growth model predicts small growth defects induced by protein misfolding. (**A**) To select the set of proteins that best correlate with growth, we chose an *R* ^2^ cutoff of 0.367, or 0.371 for a set that excludes UPR-cyto proteins (inset). (**B**) The predicted growth differences between paired strains, when each pair is held out from the dataset used to calibrate the proteomic growth model, fall within one standard deviation of each experimental measurement [4]. The UPR-cyto excluding model generates similar predictions (inset). Both models’ predictions have smaller standard deviations (vertical error) than growth measurements (horizontal error).

To assemble a set of proteins whose levels predict relative growth rate, we regressed relative protein abundance on growth-rate difference (Figure **1B**), retained proteins measured across three or more strain pairs, sorted these proteins by goodness-of-fit (*R* ^2^) values, and used slopes from the best fitting proteins to predict the growth differences between paired strains (Figure **1C**). Prediction error, the square root of the sum of the squared deviations between the predicted and measured growth differences across all five strain pairs, sharply increases when we include more than 53 proteins (Figure **2A**). Therefore, we use the 53 proteins for which *R* ^2^ ≥ 0.367 to predict growth (Table **2**). Previous models use abundance measurements from a comparable number of transcripts (72) to predict growth rate [6,13].

**Table 2:** Proteins with *R* ^2^ >0.367 used in the proteomic growth model.

**Systematic name**	**Protein name**	***R*^2^**	**Slope**	**Prediction error**
***YAL005C***	***SSA1***	***0.874***	***-94.234***	***0.674***
***YPL240C***	***HSP82***	***0.867***	***-97.145***	***0.758***
***YPL106C***	***SSE1***	***0.862***	***-32.593***	***0.718***
***YOR027W***	***STI1***	***0.856***	***-62.433***	***0.738***
***YLL024C***	***SSA2***	***0.827***	***-57.682***	***0.778***
***YMR186W***	***HSC82***	***0.822***	***-42.421***	***0.702***
***YLR109W***	***AHP1***	***0.764***	***-37.615***	***0.686***
***YNL036W***	***NCE103***	***0.732***	***-113.819***	***0.682***
YMR105C	PGM2	0.729	-101.156	0.551
***YNL007C***	***SIS1***	***0.721***	***-47.833***	***0.567***
***YJL034W***	***KAR2***	***0.715***	***-29.130***	***0.563***
***YLR216C***	***CPR6***	***0.704***	***-42.610***	***0.553***
***YHR174W***	***ENO2***	***0.700***	***-25.260***	***0.562***
YGR103W	NOP7	0.688	44.203	0.539
***YNL064C***	***YDJ1***	***0.681***	***-40.466***	***0.548***
***YLR259C***	***HSP60***	***0.635***	***-18.809***	***0.548***
***YLL026W***	***HSP104***	***0.630***	***-61.783***	***0.556***
YGL055W	OLE1	0.624	43.007	0.560
YCL050C	APA1	0.587	-30.933	0.546
***YJR045C***	***SSC1***	***0.585***	***-17.301***	***0.544***
YDL065C	PEX19	0.567	71.816	0.519
YLR384C	IKI3	0.530	-76.373	0.513
***YCR012W***	***PGK1***	***0.523***	***-22.539***	***0.529***
YJR014W	TMA22	0.518	-47.766	0.535
YHR208W	BAT1	0.515	-41.184	0.529
YDL058W	USO1	0.503	45.423	0.529
YLR304C	ACO1	0.494	29.555	0.536
YBL099W	ATP1	0.493	21.699	0.530
YDR155C	CPR1	0.488	-23.302	0.534
YPL004C	LSP1	0.483	24.783	0.531
YPL061W	ALD6	0.471	-16.504	0.528
***YAL003W***	***EFB1***	***0.465***	***-19.383***	***0.532***
YMR145C	NDE1	0.465	21.738	0.534
YGR218W	CRM1	0.464	66.649	0.513
YLR438W	CAR2	0.461	-121.160	0.500
YMR318C	ADH6	0.458	47.832	0.497
YOL111C	MDY2	0.453	-77.503	0.489
YHR010W	RPL27A	0.453	41.759	0.488
YLR249W	YEF3	0.441	9.492	0.486
YGL009C	LEU1	0.438	-50.642	0.493
YPR191W	QCR2	0.426	24.211	0.497
YER125W	RSP5	0.418	42.054	0.497
YNL281W	HCH1	0.417	-28.019	0.496
YLR056W	ERG3	0.415	47.207	0.495
YGR244C	LSC2	0.409	29.602	0.503
YNL055C	POR1	0.407	24.148	0.510
YAL060W	BDH1	0.402	-59.515	0.506
***YDR214W***	***AHA1***	***0.400***	***-32.968***	***0.498***
YKL182W	FAS1	0.392	8.636	0.498
YHR183W	GND1	0.391	15.213	0.496
YLR432W	IMD3	0.384	34.293	0.502
YNL141W	AAH1	0.376	-36.491	0.501
YDL052C	SLC1	0.367	48.201	0.502

The relative abundances of these proteins correlate (or anticorrelate) with growth in strains expressing misfolded proteins. Slopes are adjusted by a correction factor (Methods S1) that converts relative to absolute growth rates in order to allow direct comparison with previously obtained slopes [6]. Proteins highlighted in bold italics are also induced in the UPR-cyto.

To test the accuracy of our technique, we repeat the above procedure five times, each time holding out data from one of the strain pairs and predicting the growth rate of the held-out pair only. The predicted growth-rate differences for held-out pairs fall within one standard deviation of each experimental measurement (Figure **2B**). This technique’s precision, derived from as few as two replicate measurements per perturbation (Table **1**), exceeds that of repeated multi-day measurements of competitive fitness by flow cytometry [4]: for held-out data, the average standard deviation on predictions is 0.24% while the average standard deviation on experimental measurements is 0.41% (Figure **2B**).

To further estimate the expected error on our growth rate predictions, we reassembled the set of growth predictive proteins 100 times, each time holding out 30% of the data, regressing relative protein abundance on growth rate for the remaining 70%, sorting these proteins by goodness-of-fit (*R* ^2^) values, and using the best fitting proteins to predict the growth differences from the held-out data. The average standard deviations over 100 trials range from 0.09–0.14% per paired strain, with an average standard deviation across pairs of 0.12% (Figure **S1**), smaller than the average standard deviation on the most precise experimental measurements [2,4]. These results indicate that growth rates of greater precision can be extracted from proteomic data than from state-of-the-art competition assays. This technique’s predictions match the measured values to within experimental error (Figure **2B**), suggesting they are accurate as well as precise.

Surprisingly, the proteins for which expression best predicts growth in our study are not functionally similar to those found in previous screens for transcriptomic [6,7,14] and proteomic [14] signatures of growth rate. For example, the set of growth-predictive proteins does not include many ribosomal proteins (Table **2**; Table **S2**). Instead, 19 out of 53 growth-predictive proteins are components of the cytosolic unfolded protein response (UPR-cyto) [4,18,19], which is provoked in yeast by low-level protein misfolding (Table **2**; Table **S2**). Additionally, the proteomic response to growth perturbation by intracellular misfolding is of a greater absolute magnitude than expected given previously described growth-rate responses [6,7] (Figure **1C**; Figure **S2**; Table **S3**). These results suggest that the previously described growth-rate response (GRR) [7] is not the dominant response of cells during the misfolding stimulus, nor the most predictive of the resulting growth differences in this condition. However, the possibility remains that the GRR is not absent, but merely less-predictive than the stimulus-specific response [Hashimoto & Airoldi; ‘A linear model framework for genome-scale functional analysis’; unpublished manuscript]. We performed three additional analyses to test this possibility.

First, we assembled a set of growth-predictive proteins while excluding UPR-cyto proteins [4]. This set consists of 36 proteins (Table **S4**), accepts proteins with *R* ^2^ > 0.371 (Figure **2A** **; inset**), and is slightly less accurate than the original set at predicting growth rate; 4 of 5 predicted growth-rate differences fall within one standard deviation of each experimental measurement (Figure **2B** **; inset**). This set of growth-predictive proteins also has high precision—the average standard deviation on its predictions is 0.38—but it does not provide substantial evidence of a GRR. Gene ontology analysis demonstrates that functions enriched among GRR proteins (e.g. environmental stress response and ribosome biogenesis) [6,7,14] are not overrepresented among these 36 proteins.

To survey a greater number of proteins that correlate with growth, we performed a likelihood ratio test (LRT), approximating the probability distribution of the log-likelihood ratio statistic using Wilks’ theorem. This LRT is less stringent in that it allows monotonic (rather than strictly linear) relationships with growth, and it includes proteins that are detected in two or more (rather than three or more) strain pairs. Using a *P*-value cutoff of 0.05, we detected 163 proteins for which abundances covary with growth differences induced by protein misfolding (Table **S5**). The biological processes overrepresented among these 163 proteins have little overlap with the biological processes that comprise the GRR [7] or the related environmental stress response (ESR) [5] (Table **S6**).

The 71 proteins that positively covary with growth in this dataset are significantly enriched for aerobic respiration and oxidative metabolism, but these gene functions negatively covary with growth in previous studies where growth was limited by glucose concentration [6,7,14]. This enrichment remains when we repeat LRT while requiring proteins be detected in a minimum of 3 or 4 strain pairs (Tables **S7** & **S8**). Also inconsistent with GRR expectations, ribosome-related functions are not overrepresented among proteins that positively covary with growth.

Of 92 proteins that negatively covary with growth, only 7 (8%) are ESR-induced genes, while previous studies found greater overlap: 116/367 or 32% of growth-repressed transcripts [6] and 34/88 or 39% of growth-repressed proteins [14]. Of the top 50 most strongly growth-repressed, ESR-induced genes from table S1 in Brauer, et al. 2008, only HSP104, a UPR-cyto component, correlates with growth in this analysis.

One possible reason for the failure to detect a GRR or ESR is that expected changes in protein abundance, given the very mild growth perturbations studied here, may be too small to distinguish from experimental error. To determine the expected abundance difference for every protein in each strain pair, we inverted growth rate predictions from previous work in which the GRR was defined. Briefly, we obtained slopes from a linear regression of transcript levels on growth [7] and multiplied each slope by the growth rate difference between paired strains (quantified previously [4]) as well as by a correction factor that converts relative to absolute growth differences (see Methods S1). For any protein, if the resulting estimated difference in abundance is larger than the observed error on our replicate mass spectrometry experiments, we have the power to detect the expected protein-level response to our growth perturbation. Although we are using transcript levels to predict protein levels, multiple groups have measured a generally direct (1:1 on average, despite substantial variation between specific genes) relationship between mRNA and proteins changes [16,20].

**Figure 3:**
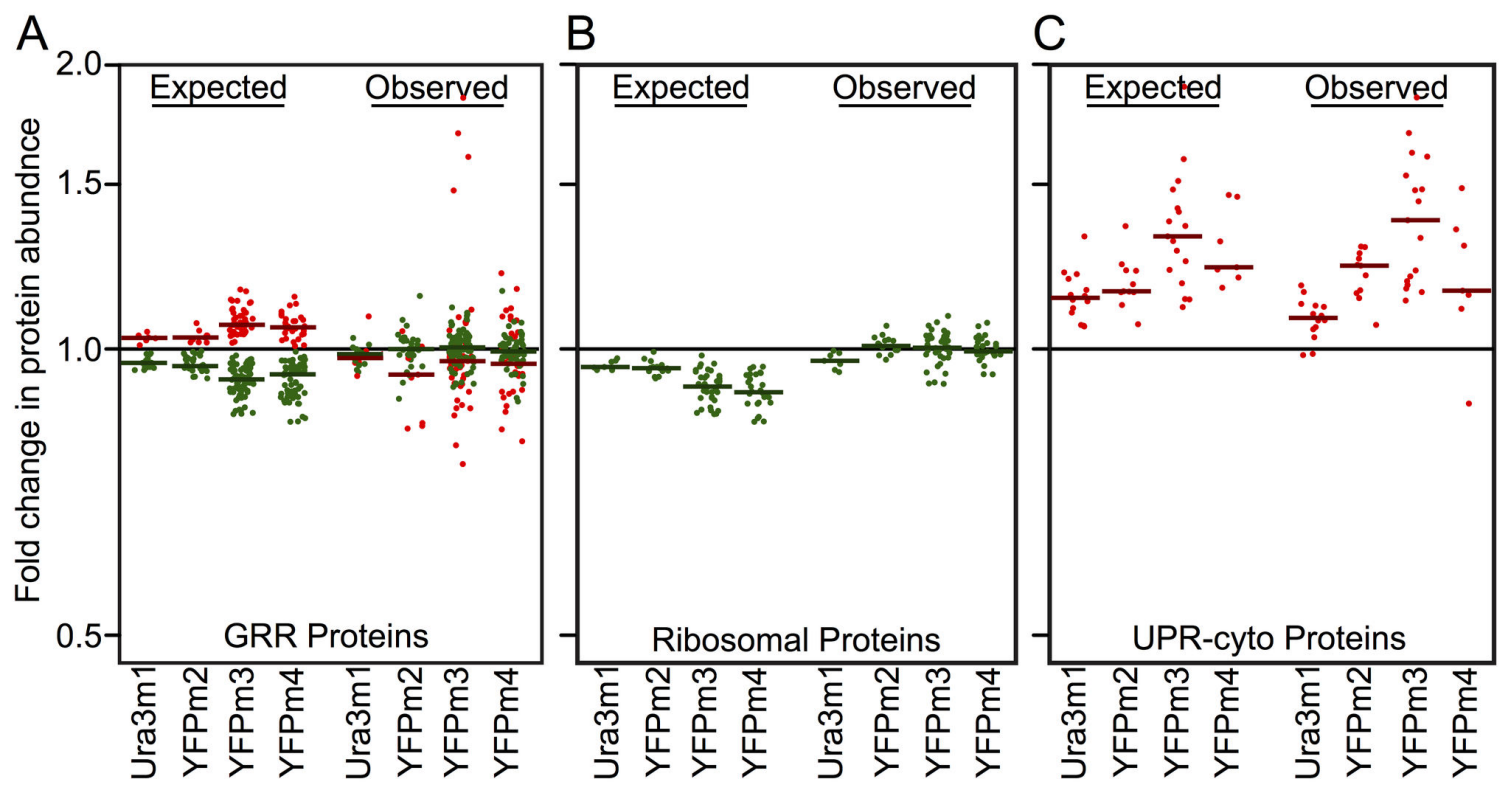
Most classes of proteins do not respond to small growth perturbations induced by protein misfolding. Expected differences in protein abundance levels are inferred from transcript data [7] (A & B) or from our proteomic data when each strain pair is held out (C), while assuming a linear response to growth. Only proteins for which we expect a significant response are plotted (i.e., proteins for which the expected fold change +/- observed error does not include 1.0 with p < 0.05). Fold changes in abundance are shown for the misfolded protein-expressing (growth-perturbed) member of each strain pair relative to the wt-expressing strain, colors represent positive (green) or negative (red) correlations with faster growth, and horizontal lines represent median values. (**A**) Most GRR proteins do not show the expected response to our growth perturbation. (**B**) Ribosomal proteins do not significantly or consistently differ in abundance between paired strains, even when the relative growth difference between strains exceeds 3% (YFPm4). (**C**) UPR-cyto protein abundance levels match expectations. Note that YFPm4 generates a smaller expected median UPR-cyto response despite inducing a larger growth defect because it contains data from only 2 replicate experiments and the most responsive proteins were not sampled.

In each set of paired strains, less than 65% of the GRR proteins expected to mount a significant response to our growth perturbations do so. Most demonstrate a weaker response than expected, while other proteins respond (some significantly) opposite expectations (Figure **3A**). Ribosomal proteins, which on the basis of GRR studies we expect to be down-regulated in slower-growing strains, also fail to show the expected response (Figure **3B**). In general, we see a poor fit between our observed protein abundances and the expectations we generated from previous studies [7] (Figure **S2**).

However, the observed changes in UPR-cyto protein levels match expectations generated from held-out data (Figure **3C**). We calculated the expected abundance change for each UPR-cyto protein in each strain pair by obtaining slopes from regressions of protein level on growth while holding out one strain pair at a time, and multiplying each slope by the growth-rate difference of the held-out pair. Using this linear model to predict protein abundances for held-out data, we were not able to predict changes in abundance for any other group of proteins (Figure **S3**). Together, the above results demonstrate that cells respond to mild growth rate differences induced by protein misfolding with a perturbation-specific response to growth.

## Discussion

Relative protein abundance measurements from strains with small differences in relative growth rates – an order of magnitude smaller than in previous studies [6,13] – reveal a set of proteins that respond linearly to growth defects. By monitoring the levels of these proteins, we can predict the growth rate of cells in batch culture to within a fraction of a percent. The proteomic model that we construct predicts growth with a precision that rivals the best growth quantitation techniques [2,4,8], requires significantly less cell culturing, and is poised to improve as mass spectrometry rapidly becomes more sensitive and higher throughput [15,16].

However, we find that proteomic growth models are not independent of the perturbations that induce growth rate differences. Proteins that predict growth in one study may not exhibit growth dependence in other conditions. This unexpected result suggests that, in yeast, growth rate is not inextricably linked to the levels of any set of proteins (not even ribosomes). The pathways that set the pace of cell division may differ depending on the growth-altering stimulus. For example, perhaps intracellular misfolding slows growth without producing the molecular signals that induce a GRR. Alternatively, it may be that a GRR (or ESR) is only induced after absolute growth drops below a threshold level.

Proteomic growth quantitation provides snapshots of how cellular content changes with tiny growth-rate adjustments. Additionally, proteomic models can estimate growth rate repeatedly over short timescales, detecting differences not only in steady-state growth rate, but also in the time needed to achieve steady-state growth after environmental insult. As technology advances, properly validated proteomic growth models might allow prediction of even smaller growth differences than can be measured using traditional techniques. The ability to study small (yet evolutionarily profound) growth differences grants access to biological regimes that have been obscured by the brutal perturbations often required to produce detectable growth effects. In these regimes, new biology surely awaits.

## Materials and Methods

### Yeast strains and growth conditions

All S288C strains were either obtained directly from a previous study [4] or are *lys2Δ* derivatives of these strains obtained via backcross to BY4742. Flasks of yeast contained 50mL growth media with either ^12^C-^14^N-lysine or ^13^C-^15^N-lysine, which was reversed in replicate experiments to exclude proteomic effects associated with stable isotope labeling. We grew cells in synthetic complete media containing 2% sucrose, 1% raffinose, and for fully induced cultures (and not uninduced cultures) 27.5 mM galactose.

### Strain pairing

In each experiment, we pair a strain expressing a wild-type protein with a strain expressing a misfolded variant of the same protein (Table **1**). Growth rate differences between these paired strains arise from differences in intracellular protein misfolding and have been quantified previously using flow cytometry [4] (Figure **1A**). All protein abundance measurements and growth rate predictions are relative to the wild-type expressing strain in each pair. Since we report relative growth rates, while previous studies describing the GRR report absolute growth rates, we convert our slopes to allow comparison with those obtained previously (Methods S1).

### Total protein isolations and quantitative proteomics

Paired strains are grown side-by-side at 30°C to log phase, after which proteins are isolated from each strain using the ball mill method [4] and then combined in a 1:1 ratio. Quantitative proteomics are performed following Geiler-Samerotte, et al.. Maximum false discovery rates (FDR) are set to 0.01 both on peptide and protein levels. From each replicate experiment, we filter proteins that are detected less than 4 times. Relaxing this requirement results in proteomic growth-models with equivalent precision and accuracy, but that utilize fewer proteins to make growth rate predictions (Table **S9**).

### Using relative protein abundance to predict relative growth rate

In order to predict growth rates we perform a similar analysis to that described in detail in Airoldi et al. 2009, with relevant changes described below and the full procedure described in detail in Methods S1. We monitor growth across 5 pairs of strains with increasing relative differences in growth rate. The growth rate prediction algorithm can be divided into three steps:

#### 1): Calibrate the proteomic growth model

For each protein for which relative abundance was measured in three or more strain pairs, we fit a linear model under heteroscedastic Gaussian error predicting relative log abundance from relative growth defect. Since our data set is unbalanced (some strain pairs are replicated more times than others), some strain pairs have more influence on the linear model than others. We improve our uncertainty estimates by using simulated samples from the parametric bootstrap (under a normal model with n=600), which allows us to give equal weight to each pair of strains as well as heteroscedasticity. Only strain pairs that meet filtering requirements – i.e. protein abundances are measured at least 4 times in at least one experiment – are included in the regression for each protein. Although using raw data predicts growth accurately for most strain pairs (Figure **S4**), simulations increase the goodness-of-fit across all proteins and the accuracy of predictions (Figure **S4** vs. Figure **2**). Therefore, regression coefficients (e.g. slope and R^2^) are fit to simulated data.

#### 2): Estimate growth-predictive proteins

We select growth-predictive proteins according to R^2^ values, selecting the R^2^ cutoff that minimizes cross-validated prediction error across all five strain pairs while allowing for the largest number of proteins to be included. The prediction error profile revealed a clear choice for the R^2^ cutoff (Figure **2A**). Prediction error is quantified as the square root of the sum of the squared deviations between the predicted and measured growth differences across all five strain pairs.

#### 3): Predict growth rates

Using the coefficient estimates from step 1, we invert the regression for each protein to obtain per-protein estimates for the mean and variance of the growth estimates and use the Gauss-Markov theorem to construct the best unbiased estimate of overall growth as a weighted average of the per-protein estimates.

### Likelihood ratio testing

We use a likelihood ratio test with p-value set to 0.05 to search for proteins which trend consistently with growth rate using the null hypothesis that abundance and growth have no relationship, and the alternative hypothesis that abundance strictly increases or decreases with respect to growth. We use Wilks’ theorem to estimate the probability distribution of the log-likelihood ratio statistic. To analyze which biological processes are up- or down-regulated with growth, we use GO::TermFinder [21].

### Calculating expected protein abundance given a GRR

To determine the expected abundance difference for every GRR protein in each pair of strains, we inverted growth rate predictions from previous work in which the universal-GRR was defined. We obtained slopes from a linear regression of transcript levels on growth [7], using slopes from glucose limited growth experiments as our strains are grown without glucose in 2% sucrose, 1% raffinose. We multiplied each slope by the growth rate difference between paired strains (quantified previously [4]) as well as by a correction factor that converts relative to absolute growth differences (see Methods S1 & Table **S10**). For any protein, if the resulting expected difference in abundance is larger than the observed error among replicate mass spectrometry experiments, we have the power to detect the expected protein-level response to our growth perturbation. For this analysis, we use only proteins that we detected in a minimum of two replicate experiments.

The expected abundance changes for UPR-cyto proteins (Figure **3C**) are not calculated using the GRR dataset [7]. Instead, these expectations are generated using data collected in this study while holding out one dataset at a time.

## Supporting Information

Figure S1 **Cross-validation of predicted growth rates was performed to estimate the expected error when inferring growth rates for a novel dataset.** Vertical error bars represent the average standard deviation of 100 cross validation experiments where 70% of proteomic data are used to fit a linear growth model and to identify a corresponding set of proteins for which abundance levels best correlate with growth. The remaining 30% of the proteomic data were used to predict relative growth rates for each strain pair. Horizontal error bars display the standard deviation on previously reported growth measurements via flow cytometry.
(PDF)

Figure S2 **The observed fold changes in protein abundance within strain pairs do not match expectations from the universal growth rate response (GRR) and are often of a larger absolute magnitude than expected.** Error bars around the observed fold changes represent 95% confidence intervals around the mean of replicate measurements. For visual purposes, we display only proteins with abundance measurements that have 95% confidence intervals smaller than 0.3. To determine the expected abundance difference for every protein within each strain pair, we inverted growth rate predictions from previous work in which the universal GRR was defined. Briefly, we obtained slopes from a linear regression of transcript levels on growth [7], and multiplied each slope by the growth rate difference between the two strains in a given pair (quantified previously [4]) and by a correction factor (see Methods S1).
(PDF)

Figure S3 **The observed fold changes in protein abundance within strain pairs do not match expectations, except for UPR-cyto proteins (black).** Error bars around the observed fold changes represent 95% confidence intervals around the mean from replicate measurements. For visual purposes, we display only proteins with abundance measurements that have 95% confidence intervals smaller than 0.3. The expected abundance changes differ from those in Figure **S2** in that they are generated from our data when the relevant dataset is held out, rather than results of previous studies. Briefly, we obtain slopes from five regressions of protein levels on growth that we performed previously while holding out one strain pair at a time, then we multiply each slope by the growth rate difference of the held out pair.
(PDF)

Figure S4 **A proteomic model constructed using unbalanced data, shown here, predicts growth less accurately than one using simulated, balanced data.** (**A**) The R^2^ values across all proteins are decreased compared to a model using balanced, simulated data (compare Figure **S4** to Figure **2**). The prediction error here is lowest when we utilize slopes from all proteins that pass filtering to predict growth rate, rather than restricting the model to use only the most predictive proteins. (**B**) The growth differences between strain pairs, each predicted by training a proteomic growth model while holding out that strain pair, do not fall all within one standard deviation of each experimental measurement [4]. Specifically, the growth rate difference between the least replicated strain pairing (YFPm4/YFPwt; n = 2) is not predicted accurately.
(PDF)

Methods S1 (DOCX)

Table S1 **This table shows all of the relative protein abundance measurements collected for this study from every mass spectrometry run.** From each replicate experiment, we include only those proteins that were detected at least four times.
(XLSX)

Table S2 **The relative abundances of these proteins correlate with growth in strains expressing misfolded proteins.** Slopes are adjusted by a correction factor (*Supplemental methods*) that converts relative to absolute growth rates in order to allow direct comparison with previously obtained slopes [6]. Proteins highlighted in gray are also induced in the UPR-cyto. Overrepresented functional categories were determined using GO::TermFinder [21].
(XLSX)

Table S3 **The slopes from our regression of protein abundance on growth rate are larger than those from a previous study in which growth was limited by glucose concentration** [6]. Our slopes are adjusted by a correction factor (see supplemental methods) that converts relative to absolute growth rates in order to allow direct comparison with previously obtained slopes [6]. For every protein we display the average number of times its abundance is measured per mass spectrometry experiment (‘ratio_count_mean’), the minimum number of times its abundance is measured per mass spectrometry experiment (‘ratio_count_min’), and the total number of experiments in which its abundance is measured at least 4 times. There are 23 total experiments (6, 4, 4, 7 and 2 for each pair of strains respectively; Table **1**).
(XLSX)

Table S4 **Proteins that best predict growth when UPR-cyto proteins are excluded.** Slopes are multiplied by a correction factor for direct comparison with those in Brauer et al. [6] The *R* ^*2*^ cutoff used here is 0.371.
(XLSX)

Table S5 **Using a likelihood ratio test with p-value cutoff set to 0.05, we find these proteins covary with growth across a minimum of two strain pairs.**
(XLSX)

Table S6 **Protein functional categories overrepresented among proteins that covary with growth.**
(XLSX)

Table S7 **Using a likelihood ratio test with p-value cutoff set to 0.05, we find these proteins covary with growth across a minimum of three strain pairs.**
(XLSX)

Table S8 **Using a likelihood ratio test with p-value cutoff set to 0.05, we find these proteins covary with growth across a minimum of four strain pairs.**
(XLSX)

Table S9 **From each replicate experiment, we filter proteins for which abundance was measured fewer than 4 times.** This does not dramatically change precision or accuracy, however, does lead to a greater number of proteins being used to predict growth.
(XLSX)

Table S10 **Cell counts and time measurements used to estimate the instantaneous exponential growth rate of the reference strain from Geiler-Samerotte et al.** [4].
(XLSX)
