# Correction: Quantifying Condition-Dependent Intracellular Protein Levels Enables High-Precision Fitness Estimates

**DOI:** 10.1371/annotation/9f5465d9-e9fa-4a80-84ca-9c9a3f6e82c7

**Published:** 2013-10-02

**Authors:** Kerry A. Geiler-Samerotte, Tatsunori Hashimoto, Michael F. Dion, Bogdan A. Budnik, Edoardo M. Airoldi, D. Allan Drummond

There was information omitted from the Funding statement. The correct version of the statement is available below.

This work was supported by National Institutes of Health grants 1R01GM088344-01 and P50GM068763. Edoardo M. Airoldi was supported by NSF CAREER IIS-1149662, NIH R01 GM096193, and a Alfred P. Sloan Research Fellowship. The funders had no role in study design, data collection and analysis, decision to publish, or preparation of the manuscript. 

