# Supplementary figures and images for: Quantifying Condition-Dependent Intracellular Protein Levels Enables High-Precision Fitness Estimates
Source: PLoS One. 2013 Sep 25;8(9):e75320. doi: 10.1371/journal.pone.0075320 (PMC3783400; doi:10.1371/journal.pone.0075320)

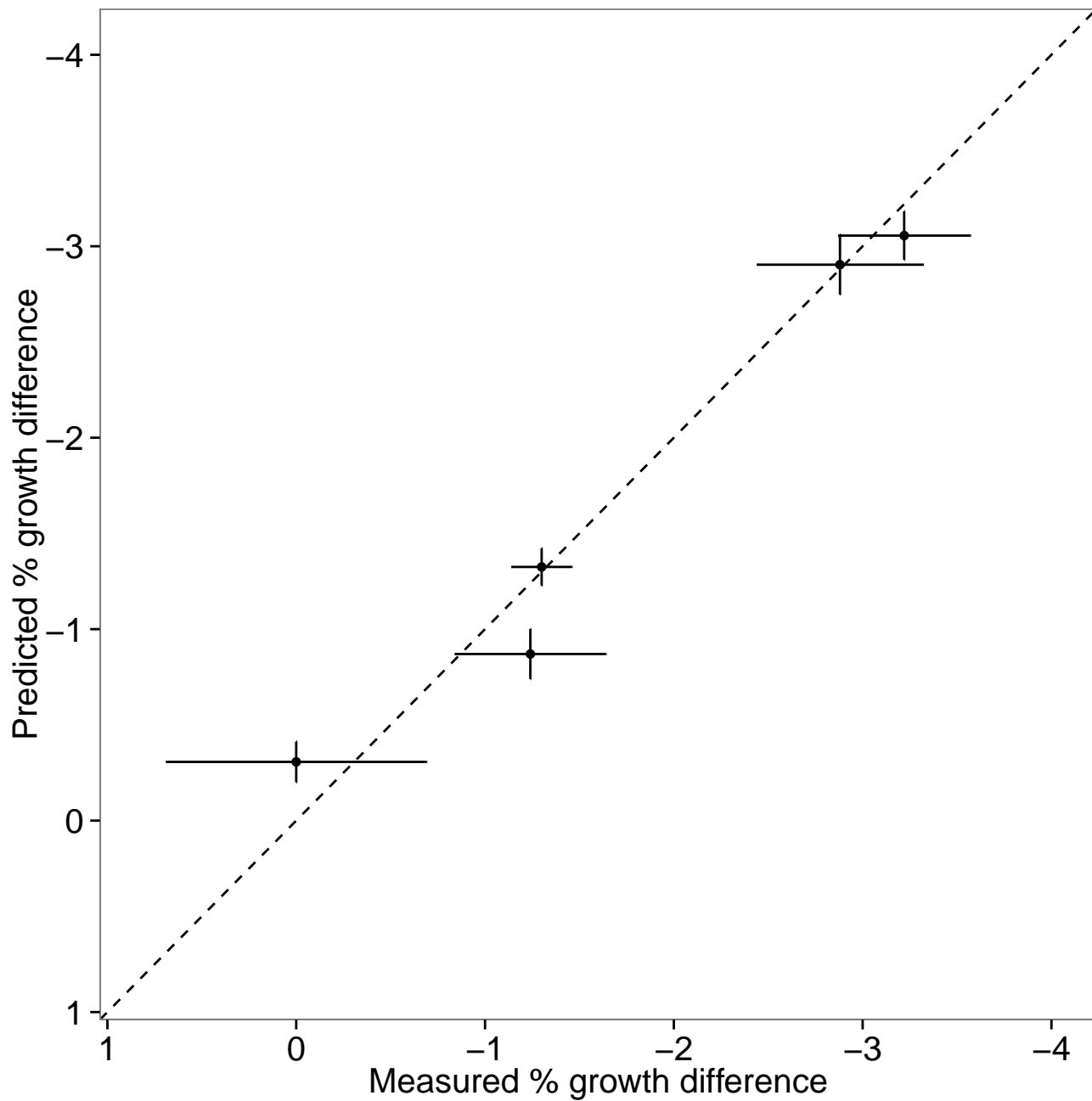

Supplement: Figure S1 — Cross-validation of predicted growth rates was performed to estimate the expected error when inferring growth rates for a novel dataset. Vertical error bars represent the average standard deviation of 100 cross validation experiments where 70% of proteomic data are used to fit a linear growth model and to identify a corresponding set of proteins for which abundance levels best correlate with growth. The remaining 30% of the proteomic data were used to predict relative growth rates for each strain pair. Horizontal error bars display the standard deviation on previously reported growth measurements via flow cytometry. (PDF) [file pone.0075320.s001.pdf]

**A**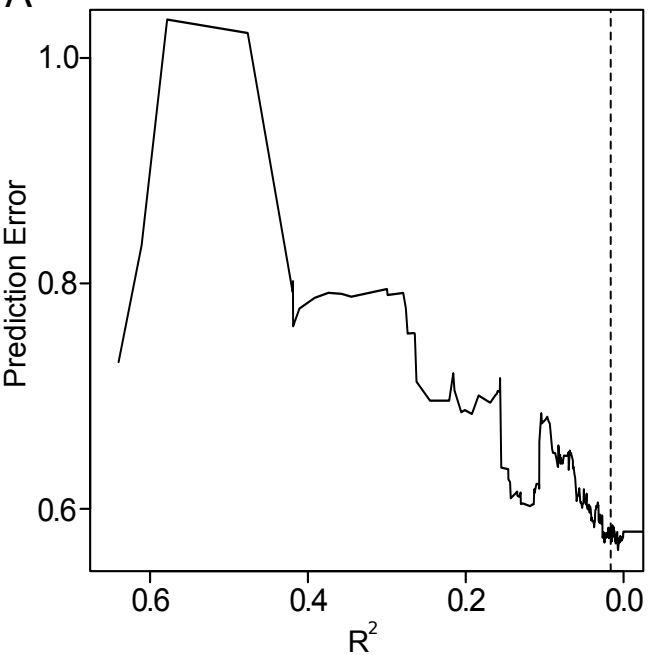**B**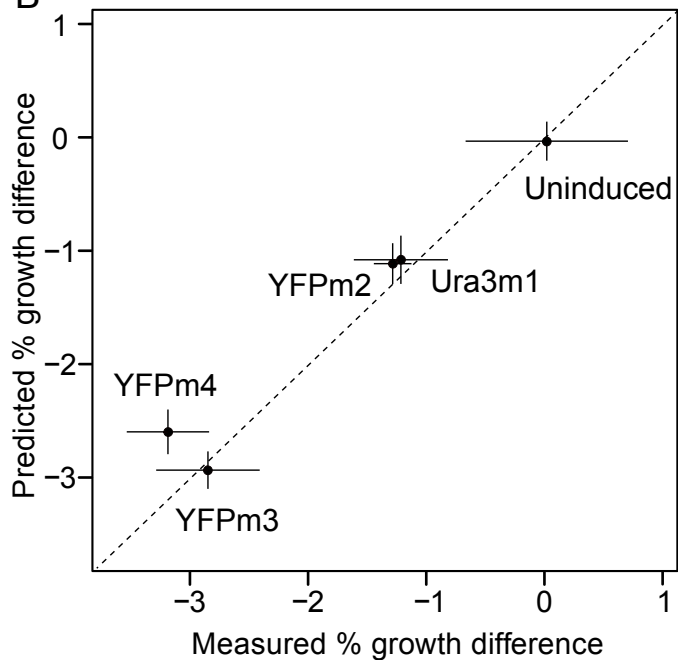

Supplement: Figure S4 — A proteomic model constructed using unbalanced data, shown here, predicts growth less accurately than one using simulated, balanced data. (A) The R2 values across all proteins are decreased compared to a model using balanced, simulated data (compare Figure S4 to Figure 2 ). The prediction error here is lowest when we utilize slopes from all proteins that pass filtering to predict growth rate, rather than restricting the model to use only the most predictive proteins. (B) The growth differences between strain pairs, each predicted by training a proteomic growth model while holding out that strain pair, do not fall all within one standard deviation of each experimental measurement [4]. Specifically, the growth rate difference between the least replicated strain pairing (YFPm4/YFPwt; n = 2) is not predicted accurately. (PDF) [file pone.0075320.s004.pdf]
