# Supplementary material for: Quantifying Condition-Dependent Intracellular Protein Levels Enables High-Precision Fitness Estimates
Source: PLoS One. 2013 Sep 25;8(9):e75320. doi: 10.1371/journal.pone.0075320 (PMC3783400; doi:10.1371/journal.pone.0075320)
Supplement: Figure S2 — The observed fold changes in protein abundance within strain pairs do not match expectations from the universal growth rate response (GRR) and are often of a larger absolute magnitude than expected. Error bars around the observed fold changes represent 95% confidence intervals around the mean of replicate measurements. For visual purposes, we display only proteins with abundance measurements that have 95% confidence intervals smaller than 0.3. To determine the expected abundance difference for every protein within each strain pair, we inverted growth rate predictions from previous work in which the universal GRR was defined. Briefly, we obtained slopes from a linear regression of transcript levels on growth [7], and multiplied each slope by the growth rate difference between the two strains in a given pair (quantified previously [4]) and by a correction factor (see Methods S1). (PDF) [file pone.0075320.s002.pdf]

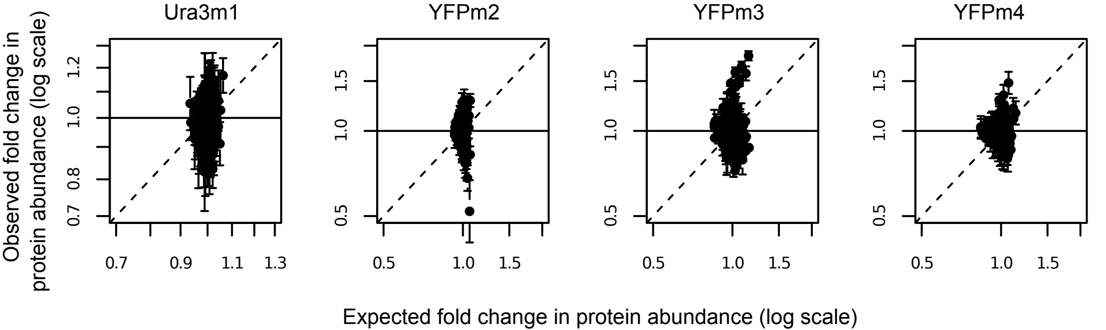
Ura3m1
YFPm2
YFPm3
YFPm4
Observed fold change in protein abundance (log scale)
Expected fold change in protein abundance (log scale)
0.7
0.8
1.0
1.2
0.9
1.1
1.3
0.5
1.5
