# Supplementary material for: Quantifying Condition-Dependent Intracellular Protein Levels Enables High-Precision Fitness Estimates
Source: PLoS One. 2013 Sep 25;8(9):e75320. doi: 10.1371/journal.pone.0075320 (PMC3783400; doi:10.1371/journal.pone.0075320)
Supplement: Figure S3 — The observed fold changes in protein abundance within strain pairs do not match expectations, except for UPR-cyto proteins (black). Error bars around the observed fold changes represent 95% confidence intervals around the mean from replicate measurements. For visual purposes, we display only proteins with abundance measurements that have 95% confidence intervals smaller than 0.3. The expected abundance changes differ from those in Figure S2 in that they are generated from our data when the relevant dataset is held out, rather than results of previous studies. Briefly, we obtain slopes from five regressions of protein levels on growth that we performed previously while holding out one strain pair at a time, then we multiply each slope by the growth rate difference of the held out pair. (PDF) [file pone.0075320.s003.pdf]

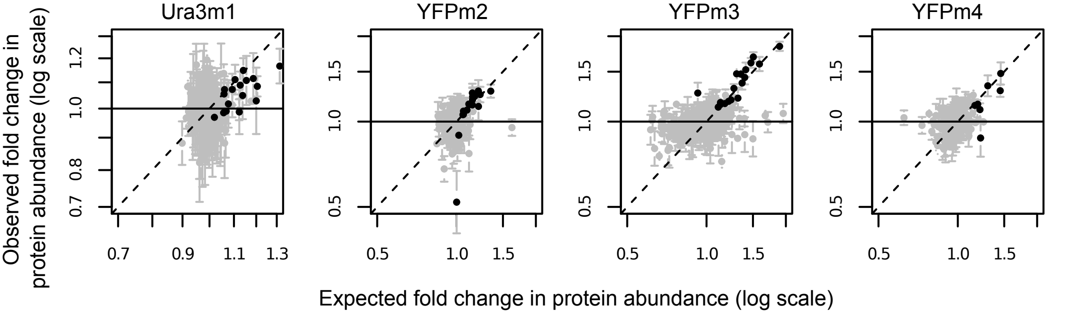

Ura3m1
YFPm2
YFPm3
YFPm4
Observed fold change in protein abundance (log scale)
Expected fold change in protein abundance (log scale)
0.7
0.8
1.0
1.2
0.9
1.1
1.3
0.5
1.5
