# Supplementary material for: Quantifying Condition-Dependent Intracellular Protein Levels Enables High-Precision Fitness Estimates
Source: PLoS One. 2013 Sep 25;8(9):e75320. doi: 10.1371/journal.pone.0075320 (PMC3783400; doi:10.1371/journal.pone.0075320)
Supplement: Methods S1 — (DOCX) [file pone.0075320.s005.docx]

# Methods

## Misfolded protein expressing yeast strains and growth conditions

All S288C strains used for mass spectrometry are derived from BY4741 or BY4742 [[1](#_ENREF_1)] and were either obtained directly from a previous study[[2](#_ENREF_2)] or are lys2Δ derivatives of these strains obtained via backcross to BY4742. Lysine auxotrophy enables stable isotope labeling for mass spectrometry. All misfolded protein expressing strains have galactose induction system modifications described in the Supplemental Data of reference [[3](#_ENREF_3)], and misfolded proteins are expressed from a smoothly inducible galactose promoter. Exponential growth rate measurements for misfolded protein expressing strains were obtained previously via fitness competitions between fluorescently tagged strains that were monitored using flow cytometry[[2](#_ENREF_2)]. We reproduced these growth conditions for misfolded protein expressing strains before harvesting cells for mass spectrometry, with some relevant modifications. Flasks of yeast contained 50mL growth media with either ^12^C-^14^N-lysine (Sigma-Aldrich) or ^13^C-^15^N-lysine (Cambridge Isotope Laboratories, Inc.) for stable isotope labeling. In each biological replicate experiment, yeast strains expressing misfolded proteins were grown side-by-side with oppositely labeled cultures expressing wild-type versions of the same protein. In each replicate experiment, flasks were equally inoculated with overnight pre-cultures and grown for ~18 hours at 30° C with shaking to a mid-log density of ~5 × 10^6^ cells/mL. As with previous growth competitions, we grew cells in SC (yeast nitrogen base, synthetic complete media) (Sunrise), containing 2% sucrose, 1% raffinose, and for fully induced cultures (and not uninduced cultures) 27.5 mM galactose. For all experiments, three mL yeast extract + peptone (YP) pre-cultures, containing 2% glucose, were inoculated with a single colony from fresh plates, and were grown overnight at 30°C with shaking before inoculating 50mL assay cultures. All experiments were repeated at least once using an isogenic independent isolate. In replicate experiments, stable isotope labeling was reversed to exclude any proteomic effects associated with growth in ^12^C-^14^N-lysine (Sigma-Aldrich) vs. ^13^C-^15^N-lysine (Sigma-Aldrich). In the case of YFPm3, one proteomic dataset differed from others in that it was separated on a PolyWAX LP column (PolyLC) and submitted in 40 fractions for mass spectrometry enabling detection of additional proteins.

## Total protein isolations

Protein isolations were performed following Geiler-Samerotte *et al* [[2](#_ENREF_2)]. Approximately 1.25×10^8^ of cells were harvested by centrifugation at 3000×*g* for 1 min from mid-log cultures. Cell pellets were washed in 1.4 mL of ice-cold 50 mM Tris-HCl, pH 8.5, and were transferred to 1.5 mL snap-cap vials. The cells were repelleted at 20,000 × *g* for 10 sec, and the wash was discarded. Cell pellets were resuspended in 50 µL of *insoluble* protein buffer (IPB; 500 mM NaCl, 50 mM Tris-HCl, pH 8.5, 1 mM PMSF, 1:100 dilution of Protease Inhibitor Cocktail Set IV (CalBiochem), 150 mM NaCl, 2% SDS and 2 mM DTT). Cell suspensions were flash-frozen upon pipette-transfer to 2.0 mL snap-cap vials that were partially submerged in liquid nitrogen and that contained a 7 mm stainless steel ball (Retsch). The cells were disrupted using a Mixer Mill MM 400 set to 30 Hz for 2×90 sec, interspersed with a brief re-cooling of the tubes in liquid nitrogen. After disruption, lysates were thawed on ice for 10 minutes.

## Quantitative proteomics

Quantitative proteomics was performed following Geiler-Samerotte *et al.*[[2](#_ENREF_2)]. Approximately 100 µg of 1:1 mixtures of ^13^C-^15^N-lysine-labeled[[4](#_ENREF_4)] and unlabeled total yeast proteins, in IPB containing 8M SDS or Urea, were digested in solution by the endoproteinase LysC using a filter-assisted sample preparation protocol[[5](#_ENREF_5)]. The resulting peptide mixtures were cleaned by C18 cartridges (3M) and run directly on an Orbitrap Velos instrument (Thermo Fisher Scientific) with 360-minute liquid chromatography and tandem mass spectrometry (LC-MS/MS) using a gas-phase fractionation procedure. Briefly: two *m/z* regions for small (350–750 *m/z*) and large (745–2000 *m/z*) ions were used to trigger MS/MS in the ion trap for the top 20 small ions from small region and top 10 large ions. Peptides were eluted with 200 nl flow rate using a NanoAcquity pump (Waters). Samples were trapped for 15 minutes with flow rate of 2 µl/min on a trapping column 100 micron ID packed for 5cm in-house with 5µm Magic C18 AQ beads (Waters) and eluted with a gradient to 20 cm 75 micron ID analytical column (New Objective) packed in-house with 3 µm Magic C18 AQ beads (Waters).

Mass spectra were analyzed using MaxQuant 1.1.1.16 [[6](#_ENREF_6)]. The data were searched against the yeast ORF database downloaded from the *Saccharomyces Genome Database* (<http://www.yeastgenome.org>) supplemented with frequently observed contaminants using the MaxQuant search engine Andromeda. Carbamidomethylated cysteines were set as a fixed modification, with oxidation of methionine and N-terminal acetylation as variable modifications. A mass deviation of 0.5 Da was set as maximum allowed for MS/MS peaks and a maximum of two missed cleavages were allowed. Maximum false discovery rates (FDR) were set to 0.01 both on peptide and protein levels. Minimum required peptide length was six amino acids. Degenerate measurements were removed by eliminating outliers greater than five standard deviations from the median relative abundance for each protein. From each replicate experiment, we filter proteins that were detected fewer than 4 times. However, allowing proteins that were only detected 1, 2, or 3 times does not dramatically change growth predictions (**Table S9**).

## Using relative protein abundance to predict relative growth rate

In order to predict growth rates we perform a similar analysis to that described in detail in Airoldi *et al.* 2009[[7](#_ENREF_7)], with relevant changes described below. We monitor growth across 5 pairs of strains with increasing relative differences in growth rate. The relative growth difference within each pair is induced by heterologous protein misfolding **(Fig. 1; Table 1)**. Briefly, the growth rate prediction algorithm can be divided into three steps:

***1) Calibrate the proteomic growth model****:* For each protein for which relative abundance was measured in three or more strain pairs, we fit a linear model under heteroscedastic Gaussian error predicting relative log abundance from relative growth defect. For each protein: given a set of strain pairs [1…n], growth measurements *x_i,j_* and replicate abundance measurements *y_i,j_* we assume that abundance measurements in strain pair i follow


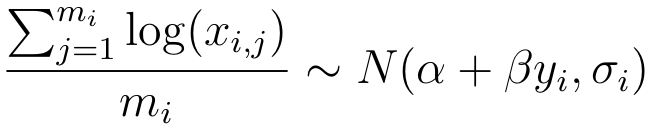


Where we set the noise per strain pair σ_i_ using a diffuse Bayesian prior,


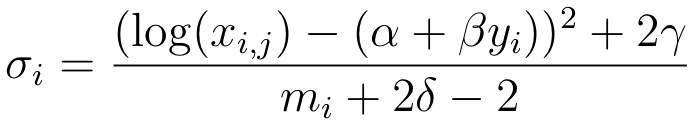


Where δ and γ are set *a priori* to be diffuse (0.505 and 0.01 respectively).

Since our data set is unbalanced (some strain pairs are replicated more times than others), some strain pairs have more influence on the linear model than others. In order to give equal weight to each pair of strains while allowing for heteroscedasticity, we simulate equal numbers (n = 600) of per-protein abundance measurements for each strain pair with mean and variance equal to those estimated from experimental data. Only strain pairs that meet filtering requirements – i.e. protein abundances are measured at least 4 times in at least one experiment – are included in the regression for each protein. Although a model using raw data predicts growth accurately for most strain pairs (**Fig S4**), simulations increase the goodness-of-fit across all proteins and the accuracy of growth predictions (**Fig S4** vs. **Fig 2**). Therefore, regression coefficients (*e.g.* slope and R^2^) are fit to simulated data.

***2) Estimate growth-predictive proteins****:* We select growth-predictive proteins according to R^2^ values, selecting the R^2^ cutoff that minimizes cross-validated prediction error across all five strain pairs. Prediction error is quantified as the square root of the sum of the squared deviations between the predicted and measured growth differences across all five strain pairs.

***3) Predict growth rates****:* Using the coefficient estimates from step 1, we invert the regression for each protein to obtain per-protein estimates for the mean and variance of the growth estimates and use the Gauss-Markov theorem to construct the best unbiased estimate of overall growth as a weighted average of the per-protein estimates.

## Comparing relative and absolute growth rates

We study relative % growth rate differences, but previous studies of transcriptional growth signatures focus on absolute growth differences. Therefore, in order to compare our findings with previous work, we derive the following conversion factor.

Let where *t*_d_ is the doubling time, so that α is the number of generations per unit time. Given instantaneous exponential growth rate μ (see below), μlog_2_*e* = α or equivalently μ = αln2. We report, as a percent, where the subscript *r* represents a reference strain that was used in the competition experiments performed in Geiler-Samerotte *et al*[[2](#_ENREF_2)] from which relative growth rates were calculated. Relative growth rates were calculated using the following formula, where the two terms on the left hand side are, collectively, the log ratios of cell counts normalized to the initial ratio. The two terms on the right hand side are the number of generations of the reference strain, α_r_t, and the growth rate difference as a fraction of the reference growth rate, , which is equal to *s*’.

Alternatively, absolute growth rates from which linear regressions of transcript level on growth were previously calculated in Brauer *et al.[*[*8*](#_ENREF_8)*]* and Slavov *et al.[*[*9*](#_ENREF_9)*]* such that

Where μ is equal to the instantaneous exponential growth rate. From this expression, it follows that

Given log_2_ gene expression fold-changes for gene *i* as at growth rate μ_2_ as compared to μ_1_, the slopes, β_x_, in Brauer *et al.[*[*8*](#_ENREF_8)*]* and Slavov *et al.[*[*9*](#_ENREF_9)*]* are calculated such that

In the cytometer/SILAC data from Geiler-Samerotte *et al.* [[2](#_ENREF_2)], given ln protein abundance fold-changes at relative growth rate *s*’_2_ compared to *s’*_1_ and assuming that these are, on average, equivalent to gene expression fold-changes, the slopes, β_a_, are:

Therefore, we need to know the growth rate of the reference strain (μ_r_ in instantaneous exponential terms) to convert competition-based relative slopes, β_a_, into chemostat-based absolute slopes, β_x_. Using cell count and time measurements from experiments performed for Geiler-Samerotte *et al*[[2](#_ENREF_2)] (**Table S10**), we estimate α_r_ = 0.0524, and μ_r_ = α_r_ *ln*(2) = 0.363, which corresponds to a doubling time of 1.91 hours.

Since we report *s*’_2_ – *s’*_1_ as a percentage, our competition-based relative slopes, β_a_, also must be multiplied by 100 to convert into chemostat-based absolute slopes, β_x_. We display converted slopes in figures and tables to allow easy comparison with previous studies.

## Likelihood ratio testing

We generalize the notion of up and down regulation to multiple strain pairs by searching for proteins which trend consistently with growth rate. Approaches such as ANOVA are not suitable for this task, since it ignores the ordering of growth rate values. Since our objective is to find proteins directly coupled with growth rate, we test for monotonicity relationships using a likelihood ratio test with p-value set to 0.05. Our null hypothesis is that mean log abundance and growth have no relationship, while our alternative is that mean log abundance either strictly increases or decreases with respect to relative growth rate. The log-likelihood statistic used for the null hypothesis is the log-likelihood of the abundance measurements across all measurements under a single normal distribution with mean and variance equal to the sample mean and variances. The statistic for the alternative hypothesis is the log-likelihood of abundance for each strain pair under a normal distribution with mean equal to the value closest to the sample mean while not violating the monotonicity constraint. We use Wilks’ theorem to estimate the probability distribution of the log-likelihood ratio statistic. To analyze which biological processes are up- or down-regulated with growth, we use GO::TermFinder[[10](#_ENREF_10)].

## Calculating expected protein abundance given a GRR

To determine the expected abundance difference for every GRR protein in each strain pair, we inverted growth rate predictions from previous work in which the universal-GRR was defined. We obtained slopes from a linear regression of transcript levels on growth[[9](#_ENREF_9)], using slopes from glucose limited growth experiments as our strains are grown without glucose in 2% sucrose, 1% raffinose. We multiplied each slope by the growth rate difference between the two strains in every pair (quantified previously[[2](#_ENREF_2)]) as well as by a correction factor that converts relative to absolute growth differences (see above). For any protein, if the resulting estimated difference in abundance is larger than the observed error on our replicate mass spectrometry experiments, we have the power to detect the expected protein-level response to our growth perturbation. For this analysis, we can use only proteins that were detected in a minimum of two replicate experiments.

To compute expected protein abundance changes for protein *i*, assuming a chemostat/microarray slope of and a change in relative growth rate from *s*’_1_ to *s*’_2_, we have:

For example, HSP*104*’s slope in the chemostat/microarray data from Slavov *et al.*[[9](#_ENREF_9)] is -11.22. Thus, given an observed relative growth rate difference of *s*’_2_ – *s’*_1_ = -0.0288 – 0 = -0.0288 (measured from strains expressing YFPm3 and YFPwt)[[2](#_ENREF_2)], we would expect, given chemostat/microarray data, to observe a protein abundance fold-change of

**References**

1. Brachmann CB, Davies A, Cost GJ, Caputo E, Li J, et al. (1998) Designer deletion strains derived from Saccharomyces cerevisiae S288C: a useful set of strains and plasmids for PCR-mediated gene disruption and other applications. Yeast 14: 115-132.

2. Geiler-Samerotte KA, Dion MF, Budnik BA, Wang SM, Hartl DL, et al. (2011) Misfolded proteins impose a dosage-dependent fitness cost and trigger a cytosolic unfolded protein response in yeast. Proc Natl Acad Sci U S A 108: 680-685.

3. Ingolia NT, Murray AW (2007) Positive-feedback loops as a flexible biological module. Curr Biol 17: 668-677.

4. Ong SE, Blagoev B, Kratchmarova I, Kristensen DB, Steen H, et al. (2002) Stable isotope labeling by amino acids in cell culture, SILAC, as a simple and accurate approach to expression proteomics. Mol Cell Proteomics 1: 376-386.

5. Wisniewski JR, Zougman A, Nagaraj N, Mann M (2009) Universal sample preparation method for proteome analysis. Nat Methods 6: 359-362.

6. Cox J, Mann M (2008) MaxQuant enables high peptide identification rates, individualized p.p.b.-range mass accuracies and proteome-wide protein quantification. Nat Biotechnol 26: 1367-1372.

7. Airoldi EM, Huttenhower C, Gresham D, Lu C, Caudy AA, et al. (2009) Predicting cellular growth from gene expression signatures. PLoS Comput Biol 5: e1000257.

8. Brauer MJ, Huttenhower C, Airoldi EM, Rosenstein R, Matese JC, et al. (2008) Coordination of growth rate, cell cycle, stress response, and metabolic activity in yeast. Mol Biol Cell 19: 352-367.

9. Slavov N, Botstein D (2011) Coupling among growth rate response, metabolic cycle, and cell division cycle in yeast. Molecular biology of the cell 22: 1997-2009.

10. Boyle EI, Weng S, Gollub J, Jin H, Botstein D, et al. (2004) GO::TermFinder--open source software for accessing Gene Ontology information and finding significantly enriched Gene Ontology terms associated with a list of genes. Bioinformatics 20: 3710-3715.
